# Supplementary material for: Deep learning-based differential gut flora for prediction of Parkinson’s
Source: PLoS One. 2025 Jan 7;20(1):e0310005. doi: 10.1371/journal.pone.0310005 (PMC11706413; doi:10.1371/journal.pone.0310005)
Supplement: Attachment — Submitted filename: Response to Reviewers.docx [file pone.0310005.s002.docx]

Dear Editor：

Thank you for your letter and the reviewers' comments on our manuscript, titled "A Deep Learning-Based Approach for Predicting Parkinson's Disease Using Differential Gut Microbiota" (Manuscript ID: PONE-D-24-12205). We appreciate the time and effort that you and the reviewers have invested in providing feedback on our manuscript. We are grateful for the insightful and valuable suggestions for improvement. These comments have been instrumental in refining our paper and will also guide our future research. We have carefully considered these suggestions and made the necessary corrections, which we hope will be approved. The changes in the manuscript are highlighted, with the revisions marked in red. The main corrections in the manuscript and our responses to each reviewer’s comments are as follows:

Response to Reviewer 1:

Dear Sumir Gandhi:

We greatly appreciate your positive feedback on our research and the issues you pointed out in this systematic review and meta-analysis. Based on your suggestions, we have made the relevant corrections in the document and have addressed your comments：

Comment1：The study has Good design and a novel study but the study involves human specimens and requires an Ethical clearance. Authors have not discussed the results of their study in the "Discussion" section properly. They need to elaborate the discussion to compare and focus their results.

Response: We greatly appreciate you pointing out this issue. This study, which involves human participants, has been reviewed and approved by the Ethics Committee of Xiangyang First People's Hospital. Written informed consent was obtained from the patients/participants for their participation in this study. Additionally, we have made the revisions you suggested to the discussion section of the manuscript and marked these changes accordingly.

Dear Reviewer 2:

Dear Reviewer:

We greatly appreciate your positive feedback on our study and the issues you pointed out in this systematic review and meta-analysis. Based on your suggestions, we have made the necessary corrections in the document and have addressed your comments accordingly.

Comment 1：The current title needs to be less generalized and should provide readers with an idea of the actual content of the manuscript. As the study focuses on developing a Deep learning tool, it is advised to add some words related to Deep learning in the title to make it more interesting and specific.

Response1：Thank you very much for pointing out this issue. We have revised the title of the study to include the term "deep learning." The revised title is " Deep Learning-based Differential Gut Flora for Prediction of Parkinson's"

Comment a：Secton 2.3 “In that study, a method for predicting Parkinson's disease patients using differential microbiota was implemented.” : Please quote the study and cite the reference.

Response a:Thank you very much for pointing out this issue, and we apologize for the confusion. Due to our writing error, "In this study" was mistakenly written as "In that study," causing misunderstanding. The method mentioned is the one used in our current study, and there is no external reference to cite.

Comment b:“The overall framework of the PGPM method constructed in that article was illustrated in Figure 1,”: Please quote the study and cite the reference.

Response b: Thank you very much for pointing out this issue. It may have been a mistake in our writing that caused the misunderstanding. The PGPM method is the name of the method used in our current study, and it cannot be referenced to external literature.

Comment c：Section 2.5 .“ LSIM In that study, the classifier for the MDOS method was based on a combined classification strategy of LSTM-SVM” : Please quote the study and cite the reference.

Response c: Thank you very much for pointing out this issue. We apologize for the confusion caused by our writing error where "MDOS" was mistakenly used. It has been corrected to "PGPM" method, and therefore, there is no reference to cite. We sincerely apologize for any misunderstanding caused.

Comment d: Provide details about who performed the annotation of deep learning tool.

Response d: Thank you very much for pointing out the issue. This study was implemented in Python (version 3.9.12) using publicly available standard libraries, including pandas (version 1.5.2), numpy (version 1.22.4), scikit-learn (version 1.2.0), torch (version 1.12), and matplotlib (version 3.6.2). We have also added this information to the manuscript.

Comment e: How was blinding performed to avoid bias?

Response e: Thank you very much for pointing out this issue. In this study, the data were collected from Parkinson's patients and their healthy spouses in the same region, with a gender ratio of 1:1. During the experiments, we utilized the DataLoader method to randomly shuffle the sample order in the dataset at the beginning of each epoch to avoid bias.

Comment f: This experiment conducted comparative trials across multiple models, with identical hyperparameter settings as follows: the number of training epochs was set to 300, the initial learning rate was set at 0.001, the batch size was set to 6,” : please cite the reference.

Response f: Thank you very much for pointing out this issue. We have added references to the manuscript as suggested. However, some of the parameters are based on our experimental experience and thus cannot be referenced.

Comment g:“optimization algorithm used was Adaptive Moment Estimation (Adam)” : Provide details of company and Version of the software.

Response g:Thank you very much for pointing out this issue. The GPU used for training in this study is the NVIDIA GeForce GTX1060 laptop GPU, with 16GB of memory and 1280 CUDA cores. We have already added this information to the manuscript.

Comment 3：Discussion: “ Bedarf et al. reported found significant differences in the composition of the gut microbiota between Parkinson's disease patients and healthy controls” : please cite the reference.

Response 3:Thank you very much for pointing out the issue. We have now added the citation of the reference at this location.

Comment 4：Discussion can be elaborated as its too short.

Comment 4：Thank you very much for bringing up this issue. We have addressed the problem of the "Discussion" section being too brief as you pointed out, and we have made the necessary revisions, which are now indicated in the manuscript.
