# Supplementary material for: Deep learning-based differential gut flora for prediction of Parkinson’s
Source: PLoS One. 2025 Jan 7;20(1):e0310005. doi: 10.1371/journal.pone.0310005 (PMC11706413; doi:10.1371/journal.pone.0310005)
Supplement: Attachment — Submitted filename: Response to Reviewers.docx [file pone.0310005.s003.docx]

Dear Editor：

Thank you for your letter and the reviewers' comments on our manuscript, titled "A Deep Learning-Based Approach for Predicting Parkinson's Disease Using Differential Gut Microbiota" (Manuscript ID: PONE-D-24-12205R1). We appreciate the time and effort that you and the reviewers have invested in providing feedback on our manuscript. We are grateful for the insightful and valuable suggestions for improvement. These comments have been instrumental in refining our paper and will also guide our future research. We have carefully considered these suggestions and made the necessary corrections, which we hope will be approved. The changes in the manuscript are highlighted, with the revisions marked in red. The main corrections in the manuscript and our responses to each reviewer’s comments are as follows:

Response to Reviewer 1:

Dear Professor Sumir Gandhi:

We greatly appreciate your positive feedback on our research and the issues you pointed out in this systematic review and meta-analysis. Based on your suggestions, we have made the relevant corrections in the document and have addressed your comments：

Comment1：Specify aims and objective of the study.

Response1: We greatly appreciate you pointing out this issue. The core objective of this study is to develop an efficient and accurate prediction method for the early diagnosis of Parkinson's disease through an in-depth analysis of gut microbiota data. We propose a differential gut microbiota-based Parkinson's prediction method (PGPM) based on deep learning, aiming to capture the subtle differences in the gut microbiome that traditional statistical methods may miss, offering new perspectives and tools for Parkinson's disease prediction. Additionally, we have incorporated the study's aims and objectives into the abstract and introduction sections and highlighted them in red. Thank you again for pointing out this issue.

Comment2：I discussion section, authors have focussed in the predictive model but not on their results. Please elaborate on that.

Response2: We greatly appreciate you pointing out this issue. We have realized that the discussion section was overly focused on our model while neglecting our results. We have made revisions to the original text, adding a section on the results of our study to the discussion. Thank you again for your guidance on our research.

Response to Reviewer 2:

Dear reviewers2：

It is a great honour to have your approval of this work.
